# Synthesis and characterization of Al-based metal–organic framework with superior performance for adsorptive desulfurization of model fuel

**DOI:** 10.1038/s41598-025-03732-y

**Published:** 2025-06-20

**Authors:** Rodaina M., Eman M. El-Sayed

**Affiliations:** 1 Alexandria Petroleum Company, Alexandria, Egypt; 2 Chemical Engineering Department, Faculty of Engineering, Alexandria University, Alexandria, Egypt; 3 Fabrication Technology Research Department, Advanced Technology and New Materials Research Institute (ATNMRI), City of Scientific Research and Technological Applications (SRTA-City), Alexandria, 21934 Egypt

**Keywords:** Adsorptive desulfurization, Al-MOF, Kinetics, Isotherm, Environmental sciences, Environmental social sciences, Chemistry, Engineering, Materials science, Nanoscience and technology

## Abstract

Metal–organic frameworks (MOFs) have recently garnered attention as promising candidates for the effective removal of sulfur-containing compounds from liquid fuels. In this study, the potential of employing Al-MIL-53 as an adsorbent for liquid fuel desulfurization is demonstrated. Material analysis through SEM, XRD, and FTIR studies was conducted. Equilibrium between in the solution and on the adsorbent surface was successfully achieved within 1 h. Optimal operational parameters for 99% Sulfur removal were identified as a 60-min adsorption time, 50 ppm initial thiophene concentration, and 2 g adsorbent dosage. The equilibrium adsorption data is adequately represented by Freundlich isotherm (R^2^ = 0.97). The adsorption kinetics of DBT by Al-MOF followed pseudo first-order model (R^2^ = 0.99). The equilibrium (qm) of the prepared Al-MOF = 11 (mg/g).

## Introduction

One of the most pressing environmental issues today is the quality of transportation fuels produced by refineries. In particular, the presence of sulfur compounds in diesel fuel poses a significant concern, as the combustion of sulfur-rich diesel leads to the formation of sulfur dioxide and sulfate particulates. These byproducts are key contributors to acid rain and atmospheric pollution. As a result, stringent environmental regulations now require the reduction of sulfur levels in gasoline to very low concentrations (< 20 mg/L)^1,2^.

Minimizing sulfur content in diesel fuel is essential to reduce harmful emissions and comply with stringent environmental regulations. The presence of sulfur in diesel leads to the formation of sulfur dioxide (SO₂) and sulfate particulates during combustion, contributing to air pollution, acid rain, and other environmental issues^3,4^. The production of sulfuric acid, rubber, and fertilizer relies on manufacturing processes involving elemental sulfur^5^. Additionally, synthetic chemistry benefits from the versatile properties of elemental sulfur. Refineries are currently grappling with the challenge of producing cleaner fuels, with the European Union imposing limits on diesel sulfur content since 2005 and Japan implementing similar restrictions in 2007. Therefore, developing simple and efficient methods for sulfur removal from fuel in the country is crucial^6^.

Traditionally, hydro-desulfurization (HDS) processes have been employed to remove sulfur from hydrocarbon fuels. This method involves converting sulfur to hydrogen sulfide under high temperature and pressure conditions with the use of noble catalysts. However, HDS is associated with high operating costs and limitations in addressing specific sulfur compounds like benzothiophenes (BTs) and dibenzothiophene (DBTs)^7^. Commonly, hydrodesulfurization processes utilize Co-MO/Al_2_O_3_ or Ni-Mo/Al_2_O_3_ catalysts at elevated temperatures (300–340°C) and pressures (20–100 atm. of H_2_)^8,9^.

In recent times, adsorptive desulfurization has emerged as a promising alternative to HDS. Adsorptive desulfurization (ADS) refers to a process in which sulfur compounds, typically found in fuels like gasoline and diesel, are removed by adsorption onto a material, usually a solid adsorbent. This method is primarily used to reduce sulfur content in fuels, which is crucial for meeting environmental regulations and minimizing the emission of harmful pollutants such as sulfur dioxide during combustion^10^. In this process, adsorbents like metal–organic frameworks (MOFs), activated carbon, and zeolites are used to capture and remove sulfur species (such as thiols, sulfides, and thiophenes) from the fuel^1^. The adsorbent’s high surface area, porosity, and the presence of functional groups (like unsaturated metal sites in MOFs) enhance its ability to adsorb sulfur compounds, making the process highly effective^11^. Numerous studies have demonstrated the potential of metal–organic frameworks (MOFs) or MOF-derived catalysts for efficient adsorptive desulfurization (ADS), showcasing impressive performance^2^. MOFs and MOF-derived catalysts represent a highly promising approach to adsorptive desulfurization due to their unique combination of high surface area, tunable porosity, and active sites for sulfur adsorption and transformationAmong various adsorbents, metal–organic frameworks (MOFs) have gained significant attention owing to their tunable porosity, high surface area, and functional versatility. For example, Zr-based MOFs have been engineered to enhance adsorption performance in environmental and fuel purification applications^12^. Similarly, cutting-edge MOF structures have shown high efficiency in adsorptive removal of pharmaceutical contaminants^13^, while MOF-based composites have proven effective in capturing heavy metals such as mercury from aqueous solutions^14^. The development and optimization of these materials hold great potential for improving the efficiency and cost-effectiveness of desulfurization processes, making them key players in the quest for cleaner fuels and reducing environmental pollution^15,16^.

Despite the growing body of research on MOFs, relatively few studies have focused on Al-based MOFs, such as Al-MIL-53, for sulfur removal in liquid fuel systems. Most existing studies emphasize the use of Zr, Fe, or Cu-based MOFs, while Al-MOFs remain underexplored, especially in terms of their adsorption behavior toward thiophene at low concentrations. Moreover, limited data is available on the kinetic and isotherm modeling of thiophene adsorption using Al-MOFs under optimized operational conditions. This lack of comprehensive evaluation represents a notable gap in the literature.

Therefore, this study aims to investigate the potential of Al-MIL-53 for liquid fuel desulfurization, highlighting its performance under optimized conditions, its fast equilibrium behavior, and its fit to adsorption models. The findings contribute to expanding the applicability of Al-based MOFs in fuel purification and provide insight into their adsorption mechanisms.

## Materials and methods

Aluminum nitrate nonahydrate (98%), terephthalic acid (≥ 98%), *N*,*N*-dimethylformamide (99.8%) were all obtained from Sigma- Aldrich., n-Hexane from UK and Thiophene (AR) from India.

### Aluminum terephthalate [MIL-53(Al)] synthesis

MIL-101(Al) was synthesized by dissolving 3 g of H₂BDC and 5 g of Al(NO₃)₃·9H₂O in 50 mL of deionized water, followed by sonication for 30 min. HNO₃ (10 mmol) was added as a modulator. The reaction mixture was then transferred to a Teflon-lined autoclave and heated at 215 °C for 12 h. After cooling, the product was washed sequentially with hot DMF (100 mL), ethanol (100 mL), and water (100 mL). Finally, the obtained powder was separated via centrifugation and dried at 100 °C for 24 h to yield MIL-101(Al)^2,17^.

### MOF characterization

X-ray powder diffraction (XRD) using Cu-Kα radiation (Shimadzu-7000, Kyoto, Japan) was employed to analyze the crystallographic phase of the prepared samples. Their morphology was examined through scanning electron microscopy (SEM, SU-70, Hitachi, Tokyo, Japan), coupled with energy-dispersive X-ray (EDX) analysis for elemental identification. The microstructure was further investigated using a JEM-2100 transmission electron microscope (TEM, JEOL, Japan). Fourier transform infrared (FTIR) analysis was performed with a Bruker ALFA spectrometer (Bruker Corporation, Ettlingen, Germany).

### Adsorptive desulfurization tests

Adsorption performance was evaluated using the batch adsorption method. Model oil was prepared by dissolving thiophene in n-hexane, with a sulfur content ranging from 50 to 100 ppm. During the adsorption experiments, varying adsorbent dosages (0.5, 1, 1.5, and 2 g) were mixed with 50 mL of the prepared model oil at different temperatures (40, 60, and 80°C) for varying time intervals of 10 min to assess the influence of these factors on sulfur reduction efficiency. The sulfur content in the samples was quantitatively analyzed using Shimadzu Multi-Type Inductively Coupled Plasma Atomic Emission Spectroscopy (ICP-AES, ICPE-9000).

The adsorption capacity of the prepared Al- MOF (Q, mg/g) and the percent sulfur reduction (R, %) were calculated using the following equations^18^:

1 $$\text{Q}=\frac{\left(\text{Co}-\text{C}\right)*\text{V}}{\text{W}}$$

2 $$\% R=\frac{(Co-Cf)}{Co}*100$$

where C_o_ and C_e_ (mg/l) are the initial concentration and final concentration of sulfur, respectively, V is the volume of the model oil, and W is the weight of the used MIL-53(Al) as an adsorbent.

### Adsorption isotherms

Equilibrium data, also referred to as adsorption isotherms, illustrate the interaction between the adsorbate and adsorbent, providing a thorough understanding of the nature of the interaction. This is crucial for optimizing the design of an adsorption system. Various isotherm equations have been developed for such analysis, with the Langmuir and Freundlich isotherms being the key models applied in this study^18,19^.

#### Langmuir’s isotherm

Langmuir’s isotherm may be used for monolayer adsorption onto a surface containing a finite number of identical sites, and assumes uniform energies of adsorption on the surface, in addition to no transmigration of the adsorbate in the plane of the surface^20^. The Langmuir isotherm model determines the maximum capacity of the adsorbent from complete monolayer coverage of the adsorbent surface.

The Langmuir isotherm^8^ is represented by the following linear equation:

3 $$\frac{{C_{e} }}{{q_{e} }} = \frac{1}{{qm\, K}} + \frac{1}{{qm }}{\text{C}}_{{\text{e}}}$$

where q_e_ is the solid-phase concentration in equilibrium with the liquid-phase concentration C_e_, q_m_ is the final sorption capacity (most commonly in mg/mg), and K is an equilibrium constant (most commonly used in l/mg). The units of K are l/mol provided that C_e_ is expressed in (mole/l).

A plot of Ce/q_e_ versus C_e_ should indicate a straight line of slope 1/qm and an intercept of 1/q_m_K.

#### Freundlich isotherm

Adsorbents that follow the Freundlich isotherm equation are assumed to have a heterogeneous surface consisting of sites with different adsorption potentials^15,21^, and each type of site is assumed to adsorb molecules, as in the Langmuir equation

4 $${\text{q}}_{{\text{e}}} = {\text{K}}_{{\text{f}}} {\text{C}}_{{\text{e}}} {\text{n}}$$

where K_f_ is constant (function of energy of energy of adsorption and temperature) and n is a constant. The freundlich isotherm was shown later to have some thermodynamic justification. Where rearranging equation above in the following form,

5 $$\ln {\text{q}}_{{\text{e}}} = \ln {\text{K}}_{{\text{f}}} + {\text{n}}\ln {\text{C}}_{{\text{e}}}$$

A straight line is obtained when plotting log q_e_ against log C_e_ which serves to evaluate the constants, K_f_ and n.

### Kinetic studies

Kinetic experiments were made by using 50 ml sulfure solutions of various concentrations (50, 75, 100 mg/l). Samples were taken at different time intervals (0–60 min) and remaining sulfure concentrations were analyzed. The rate constants were calculated using conventional rate expressions. Following formula was used to determine adsorbed sulfur amounts (q_t_)^22^:

6 $${\text{q}}_{{\text{t}}} = \left( {{\text{C}}_{{\text{o}}} - {\text{C}}_{{\text{t}}} } \right)*{\text{V}}/{\text{m}}$$

where q_t_ (mg/g) is the adsorption capacity at time t, C_o_ (mg/l) is the initial sulfur concentration, C_t_ (mg/l) is the concentration of sulfur in solution at time t, V (l) is the solution volume, and m (g) is the amount of the adsorbent.

#### Pseudo-first-order reaction kinetic

Simple linear equation for pseudo-first-order reaction kinetic is given below^7^:

7 $$\ln \left( {{\text{q}}_{{\text{e}}} - {\text{q}}_{{\text{t}}} } \right) = \ln {\text{q}}_{{\text{e}}} - {\text{k}}_{1} {\text{t}}$$

where; k_1_ is the rate constant of the first-order adsorption, q_t_ is the amount of sulfur adsorbed at time ‘t’ (mg/g) and q_e_ is the amount of sulfur adsorbed at saturation (mg/g).

#### Pseudo-second-order reaction kinetic

Pseudo-second-order reaction kinetic can be expressed as^21^:

8 $${\text{t}}/{\text{q}}_{{\text{t}}} = 1/{\text{k}}_{2} {\text{q}}_{{\text{e}}}^{2} + {\text{t}}/{\text{qe}}$$

where k_2_ (g/mg min) is the pseudo-second-order rate constant, q_e_ the amount adsorbed at equilibrium and qt is the amount of sulfur adsorbed at time ‘t’. Similar to the pseudo-first-order reaction kinetic, q_e_ and k_2_ can be determined from the slope and intercepts of plot t/q_t_ versus t.

## Results and discussions

### Characterization of the prepared MIL-53(Al)]

**Fig. 1:**
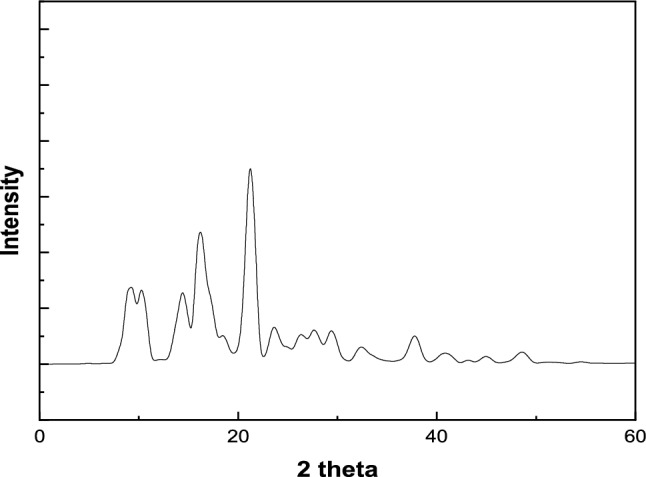
XRD Pattern of the prepared MIL-53(Al)].

Figure 1 presents the X-ray diffraction (XRD) pattern of the synthesized Al-MOF. The pattern shows well-defined, sharp peaks at specific 2θ angles, namely 7°, 10°, 13°, and 20°, which are consistent with the calculated diffraction peaks of the Al-MOF structure based on the simulation (CCDC No. 181153)^16^. These distinct and intense peaks indicate the high crystallinity and well-ordered structure of the Al-MOF, confirming that the material was effectively synthesized. The observed XRD pattern matches closely with the simulated one, further validating the successful formation of the intended metal–organic framework. The presence of these peaks suggests that the Al-MOF retains its structural integrity during synthesis and is consistent with the crystallographic properties predicted for this specific framework.

The average crystallite size of Al-MIL-53 was calculated using the Scherrer equation^19^:

9 $${\text{D}} = {\text{K}}\lambda /\left( {\upbeta \cos \uptheta } \right)$$

where D, Crystallite size (nm); K, Shape factor (typically 0.9); λ, X-ray wavelength (Cu Kα, 1.5406 Å); β, Full width at half maximum (FWHM) in radians; θ, Bragg angle (in degrees).

The estimated crystallite size is approximately 32 nm, indicating nanocrystalline domains.

**Fig. 2:**
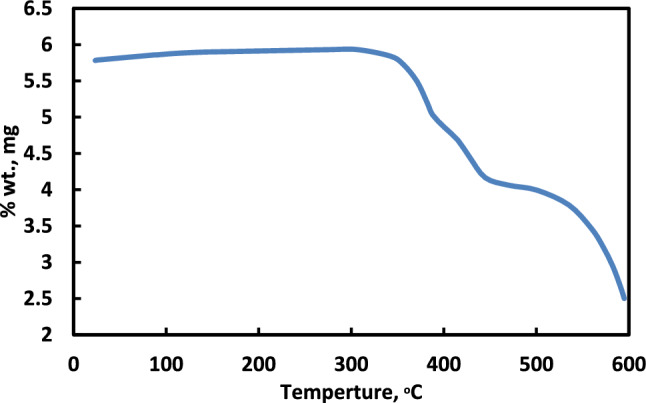
TGA curve of the prepared MIL-53(Al)].

The thermal stability of the synthesized Al-MOF was evaluated through thermogravimetric (TG) analysis, as illustrated in Fig. 2. The TG curve reveals a minimal weight loss of approximately 0.6 wt% below 200 °C, which can be attributed to the desorption of physically adsorbed water and residual solvent molecules trapped within the porous framework. This slight mass reduction indicates that the structure is largely free of volatile impurities. A significant onset of weight loss is observed around 340 °C, signifying the thermal decomposition of the organic linkers within the MOF structure. The relatively high decomposition temperature confirms that the Al-MOF exhibits excellent thermal stability, making it suitable for applications that involve elevated temperatures. Beyond this point, a continuous mass loss is recorded, which corresponds to the gradual breakdown of the framework’s organic components^23^.

**Fig. 3:**
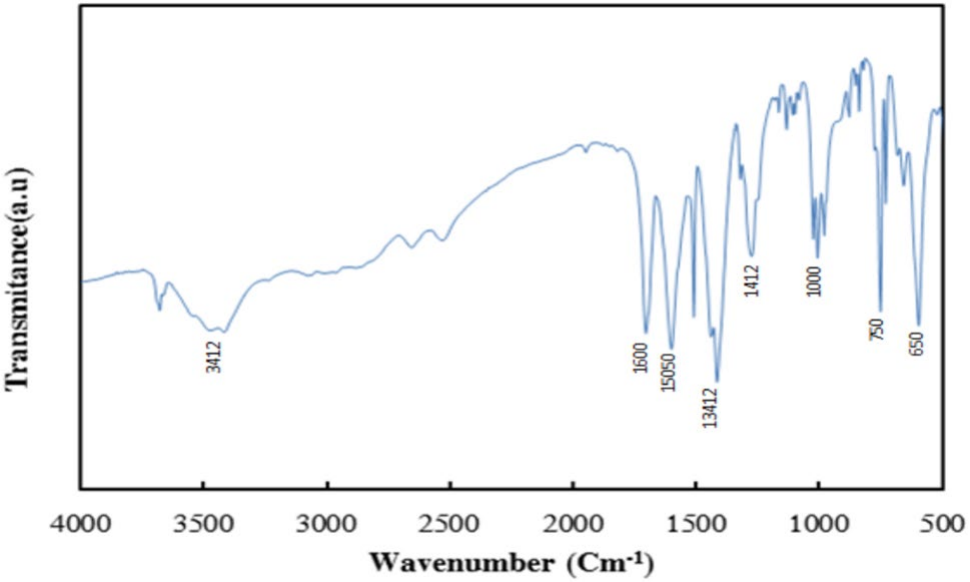
FTIR spectra of a synthesized, MIL-53(Al)].

The Fourier transform infrared (FTIR) spectrum of the Al-MOF, presented in Fig. 3, displays key characteristic peaks confirming the functional groups present. A prominent absorption band near 1600 cm^−1^ is associated with the stretching vibrations of carboxylate groups, indicating coordination with terephthalate linkers. Another noticeable band around 1450 cm^−1^ corresponds to C–C stretching vibrations. Additionally, the spectrum shows an in-plane C–H bending vibration at approximately 1000 cm^−1^, while the out-of-plane C–H bending is evident near 650 cm^−1^^2,17^.

**Fig. 4:**
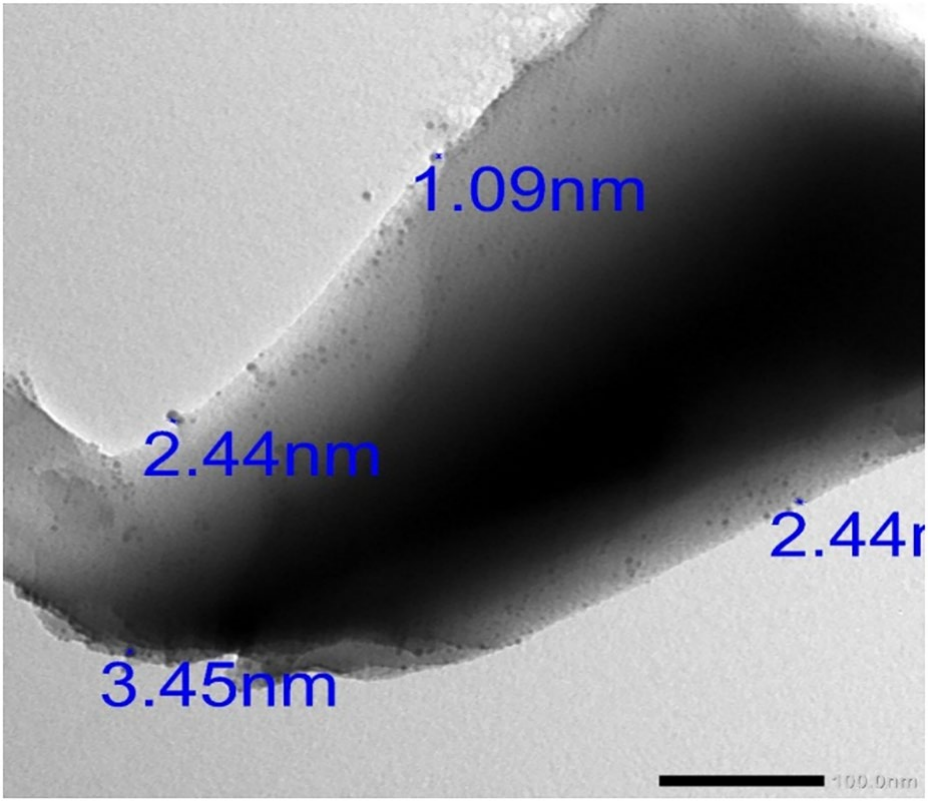
TEM images of MIL-53(Al)].

**Fig. 5:**
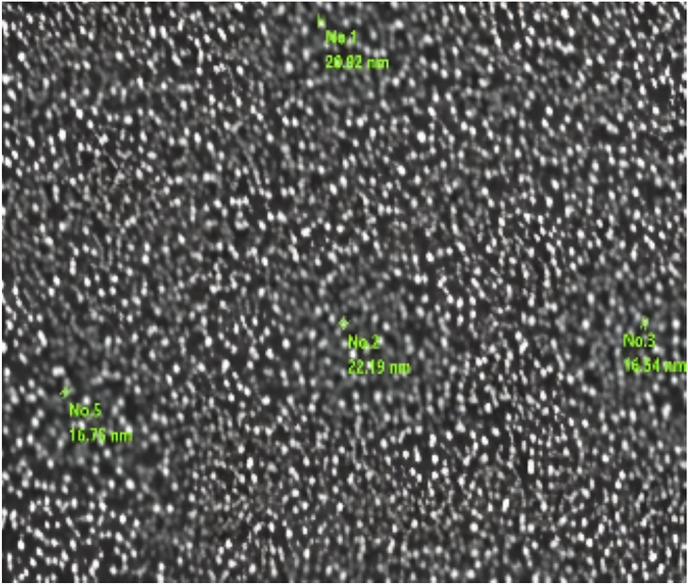
SEM images of the prepared MIL-53(Al)].

The TEM and SEM images in Figs. 4 and 5 were used to define morphometric characteristics of the prepared MOF. The TEM images showed spherical particles for the chemical method with various diameters with average diameter 18.9 nm.

The characterization of the adsorbent revealed an average pore diameter of 23.4 nm, indicating that the material falls into the mesoporous range (2–50 nm) according to IUPAC classification. Mesoporous materials are particularly suitable for the adsorption of moderately sized organic molecules like thiophene, as they offer sufficient diffusion pathways and surface accessibility.

However, despite the favorable pore size, the BET-specific surface area was low, 0.115 m^2^/g, and the total pore volume was also minimal at 0.0013 cm^3^/g. The observed high adsorption performance, it might be attributed to the Strong chemical affinity between the adsorbent surface and thiophene molecules (e.g., Lewis acid–base interaction) and the highly active functional groups present on the surface rather than reliance on surface area alone^20^.

### Desulfurization performance of Al- MOF

#### Effect of contact time

**Fig. 6:**
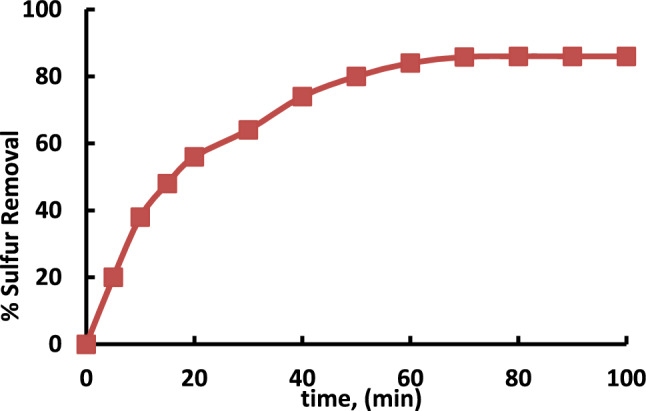
Effect of contact time on sulfur removal using Al-MOF.The experiments conditions: catalyst dose = 1 g; model oil, 50 mL; initial sulfur conc. 50 ppm, 25 °C.

The adsorption experiments were conducted by combining 0.5 g of the adsorbent with 50 mL of model oil and varying the contact time. With the interval of time, the removal of sulfur increases (Fig. 6) and reaches the equilibrium after 60 min. During the first stage, the adsorption rate was fast due to the high-concentration gradient between the sulfur molecules in the feed diesel and on the surface of the adsorbent. Therefore, a bulk transport of sulfur molecules from the solution onto the MIL-53(Al)] surface takes place. This is indicated by the plateau line and become asymptotic after 60 min signifying that after equilibrium is attained the rate of adsorption equals the rate of desorption^24^.

#### Initial sulfur concentration (ppm)

**Fig. 7:**
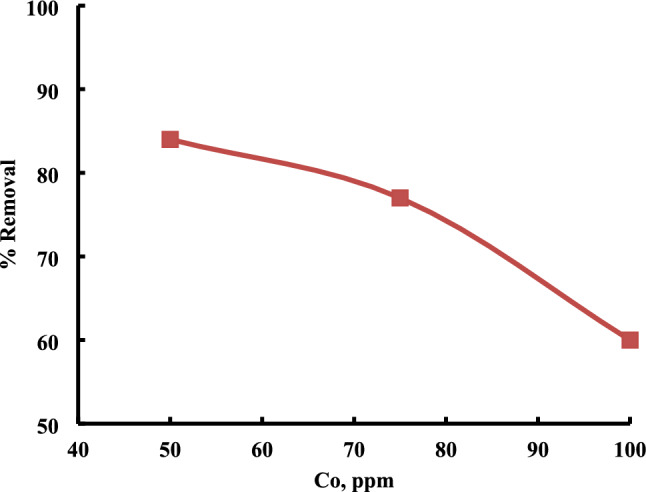
Effect of initial concentration on sulfur removal using Al-MOF. The experiments conditions: catalyst dose = 1 g; model oil, 50 mL; contact time = 60 min, 25 °C.

Generally, as the initial sulfur concentration increases, the adsorption capacity of the adsorbent tends to rise^25^. A higher initial sulfur concentration provides a greater number of sulfur species for adsorption, potentially leading to a more efficient removal of sulfur from the fuel till reaching the saturation point: There is a saturation point beyond which further increases in initial sulfur concentration not result in a proportional increase in adsorption capacity (Fig. 7)^26^.

#### Effect of adsorbent dose

**Fig. 8:**
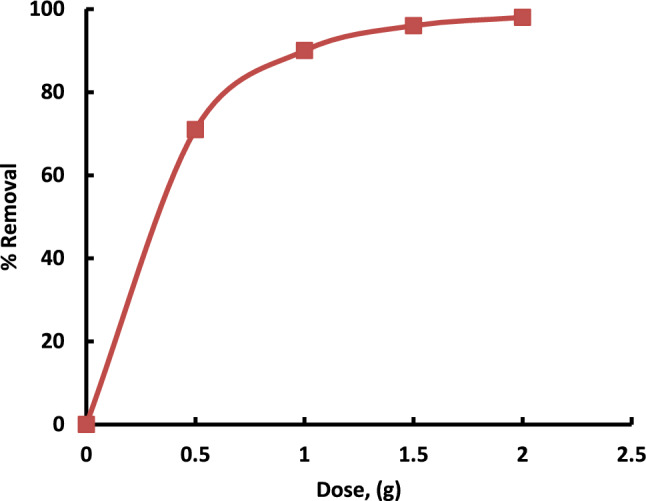
Effect of catalyst dosage on sulfur removal using Al-MOF. The experiments conditions: Initial conc. = 50 ppm; model oil, 50 mL; contact time = 60 min, 25 °C.

The influence of adsorbent amount on the sulfur reduction efficiency was studied by mixing different dosages of catalyst (0.5, 1, 1.5, and 2 gm) with 50 ml of model oil at room temperature for 20 min. The results are given in Fig. 8. It was found that the increase in adsorbent load from 0.5 to 2 g, increase the % reduction of sulfur. This may be returned to that increasing adsorbent dosage provides more surface area, more adsorption sites, and active functional groups, thereby increasing the adsorptive capacity^27^. The effect of catalyst dosage on adsorptive desulfurization is a critical factor that influences the efficiency and performance of the desulfurization process. The term “catalyst dosage” refers to the amount or concentration of the catalyst used in the adsorption system. Here are some key aspects to consider: Increasing the catalyst dosage often leads to a higher adsorption capacity for sulfur compounds. A greater amount of catalyst provides more active sites for adsorption, allowing for the removal of a higher concentration of sulfur from the fuel^28^.

#### Effects of adsorption temperature

**Fig. 9:**
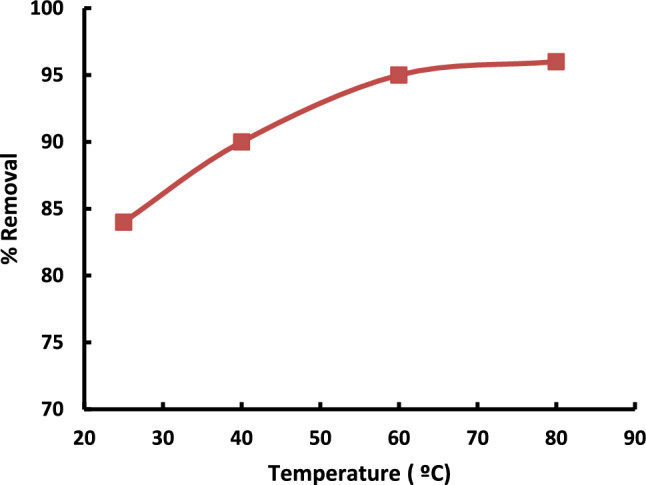
Effect of Temperature on sulfur removal using Al-MOF. The experiments conditions: (Initial conc. = 50 ppm; model oil, 50 mL; contact time = 60 min, adsorbent dosage = 1 g).

The impact of adsorption temperature on adsorptive desulfurization is substantial and plays a crucial role in determining the overall efficiency of the desulfurization process. The temperature at which adsorption takes place can affect various factors, including adsorbent-adsorbate interactions, adsorption kinetics, and the overall adsorption capacity for sulfur compounds in fuels. The results showed in Fig. 9 revealed that, an increase in adsorption temperature can enhance the adsorption capacity of the adsorbent for sulfur compounds. Elevated temperatures often contribute to improved desulfurization efficiency by facilitating the mobility of sulfur-containing molecules and enhancing their affinity for the adsorbent material^29^.

### Equilibrium isothermal adsorption

**Fig. 10:**
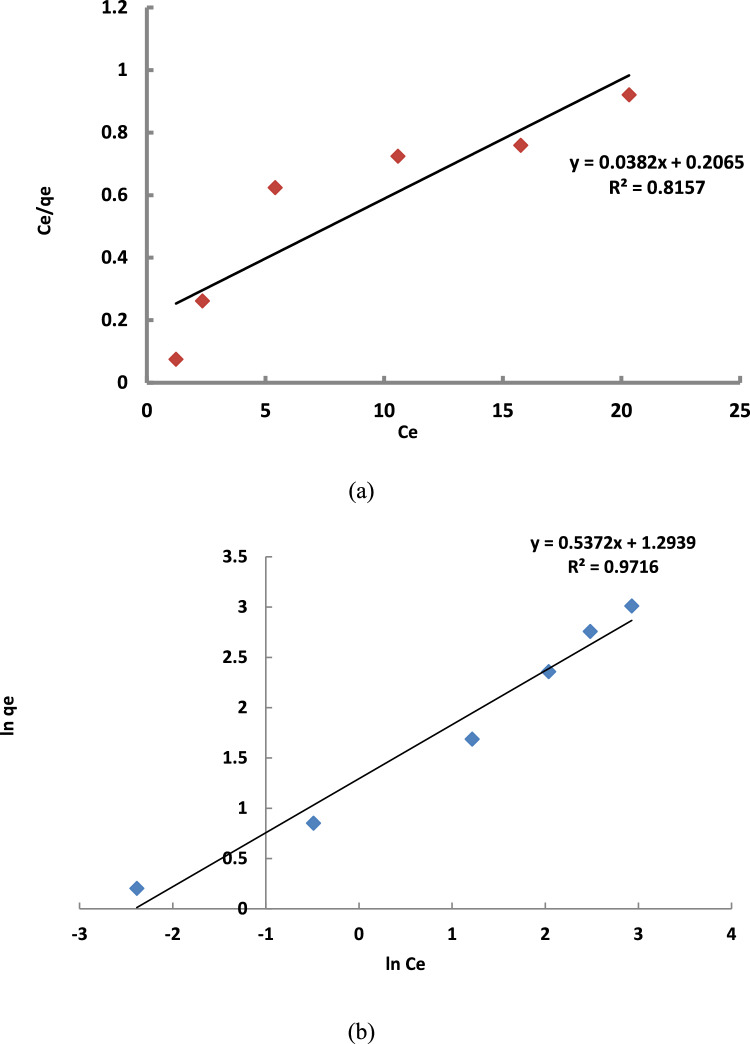
Langmuir (**a**) and Freundlich (**b**) adsorption isotherms for thiophene adsorption over the prepared Al-MIL-53.

Langmuir and Freundlich plots are shown in Fig. 10. The calculated regression coefficient values (R^2^) for the Freundlich model were higher than those for the Langmuir model, suggesting that the Freundlich model was more accurately for Al-53. This implies that the adsorption process is multlilayer adsorption on heterogeneous surfaces rather than monolayer adsorption on homogenous surface^23,26^.

### Adsorption kinetics

**Fig. 11:**
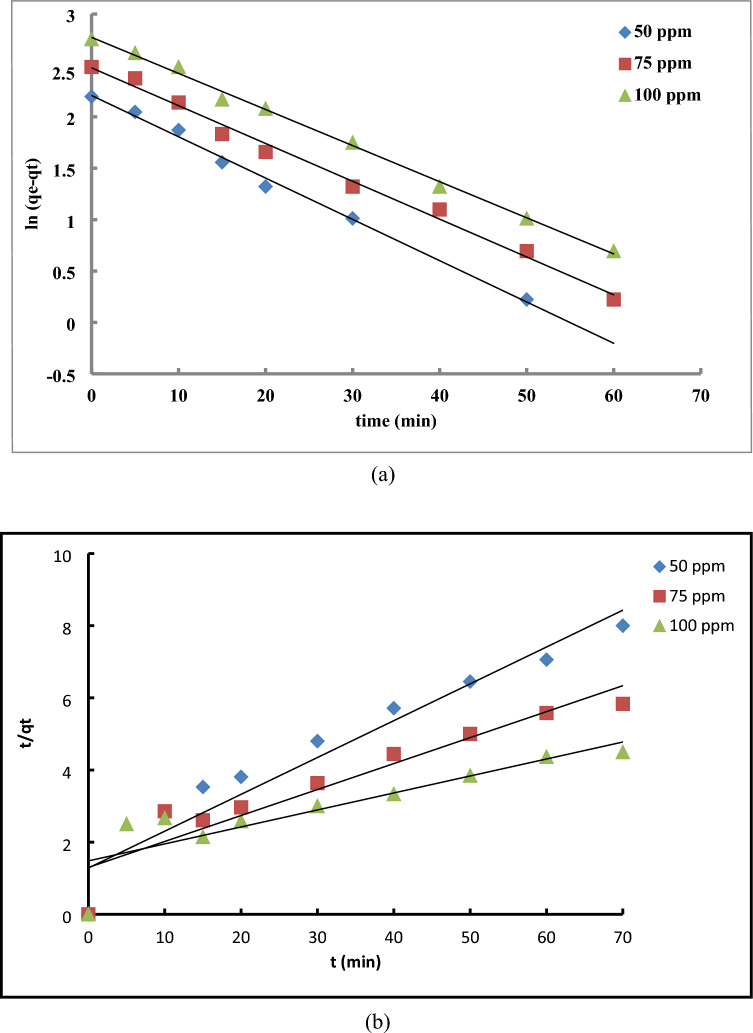
The pseudo-first-order and pseudo-second-order kinetic models for thiophene adsorption over Al-MIL-53.

To elucidate the sulfur-sorption process onto Al-MOF, the pseudo-first-order and pseudo-second-order relations were employed to fit the kinetics. As illustrated in Fig. 11, the simulated high regression coefficient values (R^2^) indicated that the adsorption process over the prepared MOF matched well with the pseudo first-order kinetic model^30^.

## Recycling of spent catalyst

**Fig. 12:**
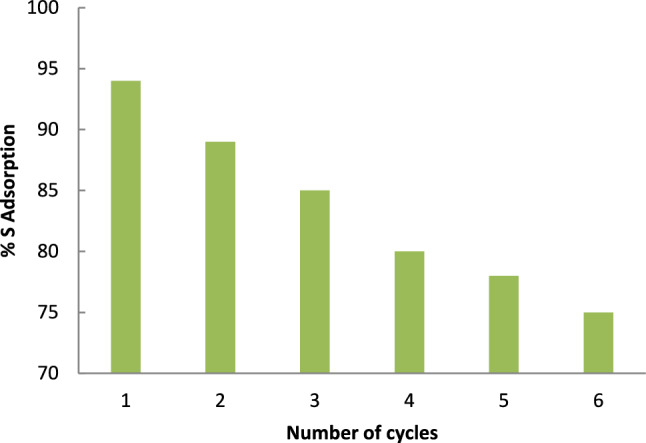
Effect of recycling of the used catalyst on the sulfur removal in the oxidative desulfurization.

After each experiment, the spent catalyst was separated by centrifugation, followed by washing with acetonitrile to remove the adsorbed oxidation product (sulfone). The catalyst was then regenerated by vacuum drying for 24 h. The regenerated catalyst was reused in subsequent cycles for thiophene removal, using the optimized operating parameters for the desulfurization process. As shown in Fig. 12, the catalyst efficiency declined to 75% after six cycles.

The Al-based MOF in this study shows the highest removal efficiency for thiophene among all listed materials as shown in Table 1. This suggests that it has greater affinity, favorable pore structure, and possibly stronger interactions (e.g., π-complexation or Lewis acid–base) with thiophene molecules.

**Table 1:** The removal efficiency for thiophene using different materials.

Adsorbent	Initial thiophene concentration (ppm)	Removal efficiency (%)	Contact time (min)	References
Al-based MOF (This study)	50	99%	60	Current study
Activated Carbon	50	71%	90	^31^
Zn-MOF	50	84%	60	^32^
ZIF-8 MOF	50	86%	60	^33^
Ni/Al₂O₃	50	75%	120	^34^
Clay-based adsorbent	50	60%	120	^35^

## Adsorption mechanism

**Fig. 13:**
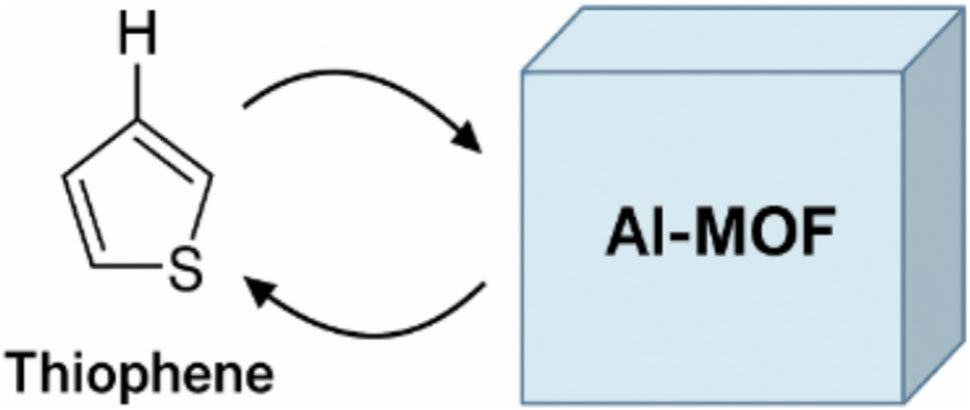
Thiophene adsorption mechanism on the prepared Al-MOF.

The adsorption of thiophene onto Al-based metal–organic frameworks (Al-MIL-53) primarily occurs through a combination of physical and chemical interaction see Fig. 13. The aromatic ring of thiophene interacts with the benzene rings in the terephthalate linkers of the MOF through π–π stacking. Thiophene, being an electron-rich aromatic compound, can act as a donor and interact with electron-deficient sites in the framework. In addition the aluminum metal centers in the MOF act as Lewis acid sites, which can interact with the sulfur atom in thiophene, a Lewis base. This creates a strong coordination bond and enhances selectivity toward sulfur-containing compounds. Also, the hydrophobic character of the MOF surface can favor the adsorption of non-polar compounds like thiophene over polar impurities, increasing selectivity and capacity.

## Conclusion

The current investigation demonstrates the viability of utilizing Al-MIL-53 as an adsorbent for the desulfurization of liquid fuels. SEM, XRD, and FTIR studies were conducted to analyze the prepared material. Equilibrium between thiophene in the solution and on the adsorbent surface was effectively established within 1 h. The optimal operational parameters were determined as 60 min of adsorption time, 50 ppm initial thiophene concentration, and 1 g of adsorbent dosage. Freundlich isotherm best represented the equilibrium adsorption data at all temperatures. This implies that the adsorption process is multilayers adsorption on heterogeneous surface. The adsorption process over the prepared MOF matched well with the pseudo first-order kinetic model. Al-MOF-based materials provide an efficient and sustainable approach to removing dibenzothiophene (DBT) from fuels, offering high adsorption capacity, selectivity, and potential for regeneration. This makes them a promising solution for adsorptive desulfurization in the petroleum industry.

## Data Availability

The datasets used and/or analysed during the current study available from the corresponding author on reasonable request.
